# Tri-specific tribodies targeting 5T4, CD3, and immune checkpoint drive stronger functional T-cell responses than combinations of antibody therapeutics

**DOI:** 10.1038/s41420-025-02329-8

**Published:** 2025-02-10

**Authors:** Margherita Passariello, Lorenzo Manna, Rosa Rapuano Lembo, Asami Yoshioka, Toshikazu Inoue, Kentaro Kajiwara, Shu‑ichi Hashimoto, Koji Nakamura, Claudia De Lorenzo

**Affiliations:** 1 Department of Molecular Medicine and Medical Biotechnologies, University of Naples “Federico II”, Naples, Italy; 2 Ceinge - Biotecnologie Avanzate S.C. a.R.L, Naples, Italy; 3 European School of Molecular Medicine, University of Milan, Milan, Italy; 4 Chiome Bioscience Inc, Tokyo, Japan

**Keywords:** Translational research, Cancer immunotherapy

## Abstract

One of the most promising cancer immunotherapies is based on bi-specific T-cell engagers (BiTEs) that simultaneously bind with one arm to a tumor-associated antigen on tumor cells and with the other one to CD3 complex on T cells to form a TCR-MHC independent immune synapse. We previously generated four novel tri-specific tribodies made up of a Fab targeting 5T4, an oncofetal tumor antigen expressed on several types of tumors, a scFv targeting CD3 on T cells, and an additional scFv specific for an immune checkpoint (IC), such as PD-1, PD-L1 or LAG-3. To verify their advantages over the combinations of BiTEs (CD3/TAA) with IC inhibitors, recently used to overcome tumor immunosuppressive environment, here we tested their functional properties in comparison with clinically validated mAbs targeting the same ICs, used alone or in combination with a control bi-specific devoid of immunomodulatory scFvs, called 53 P. We found that the novel tri-specific tribodies activated human peripheral blood mononuclear cells more efficiently than clinically validated mAbs (atezolizumab, pembrolizumab, and relatlimab) either used alone or in combination with 53 P, leading to a stronger tumor cytotoxicity and cytokines release. In particular, 53L10 tribody targeting PD-L1 displayed much more potent effects than the combination of 53 P with all the clinically validated mAbs and led to complete tumor regression in vivo, showing much higher efficacy than the combination of 53 P and atezolizumab. We shed light on the molecular basis of this potentiated anti-tumor activity by evidencing that the insertion of the anti-PD-L1 moiety in 53L10 led not only to stronger binding of the tri-specific to tumor cells but also efficiently blocked the effects of increased PD-L1 on tumor cells, induced by IFNγ secretion also due to T-cell activation. These results are important also for the design of novel T-cell engagers targeting other tumor antigens.

## Introduction

Tumor therapy is confronted with continuous challenges, where the main goals are still represented by higher specificity, efficacy, and lower side effects compared to those of conventional chemotherapy and radiotherapy [1–5].

To this aim, different immunotherapy approaches have been developed in the last decades, that can be chosen as more appropriate depending on the targeted tumor and its microenvironment. Among them, chimeric antigen receptor-T (CAR-T) cells are innovative tools that have shown great results in hematological malignancies. They consist of T cells, usually obtained from the patient, that are engineered in order to provide them with a chimeric receptor made of two main domains: an extracellular single-chain variable fragment (scFv), specific for a tumor-associated antigen (TAA), and an intracellular signaling domain, whose purpose is to activate the biochemical pathways associated with CD3. CAR-T cells have shown undeniable efficacy in the treatment of hematological tumors [6–8], however, this technology is not free of limitations, namely the expensive, complex, and long production procedures, as well as potential chronic toxic side effects [9, 10].

Another fascinating class of therapeutics that has been recently generated is based on the T-cell engager bi-specific antibodies. Their mechanism of action is simple, and yet very effective: these agents present a first binding site recognizing a TAA and a second binding site specific for a receptor on immune cells, such as CD3, CD137, or CD16 (in the case of natural killer (NK) cells) [11]. The goal of this treatment is the recruitment of immune cells to cancer cells, activation of T-cell proliferation pathways, and consequent enhancement of their ability to kill tumors. The very first bi-specific T-cell engager (BiTE) approved by the food and drug administration (FDA) in 2014 was Blinatumomab, which presents binding sites for CD3 and CD19, and it has proven to be extremely effective and safe in the treatment of acute lymphocytic leukemia and non-Hodgkin lymphoma [12]. The Blinatumomab approval paved the way for the generation of several other bi-specific agents, and generally, this approach has proven to be very effective in the treatment of hematological malignancies [13–15].

In the last few years, several T-cell engagers against different types of solid tumors, such as melanoma, prostate, and ovarian cancer, have been developed and tested in clinical trials, showing promising results [13, 16]. This suggests that T-cell engagers have the potential to become valid tools to treat an even broader spectrum of oncological diseases in the near future, and further studies need to be done in order to achieve this goal [17].

During the last decade, immune checkpoint inhibitors (ICIs) have revolutionized the field of cancer immunotherapy; nevertheless, the clinical benefits obtained with the immune checkpoint (IC) blockade are limited to only a small subset of patients [18–20]. To overcome these limits, combinations of ICIs with several other anti-cancer treatments have been approved by FDA, and some other are currently being tested in clinical trials, showing remarkable success in various cancers and providing hope for the patients not responding to ICIs monotherapy [21–23]. Among ICs, programmed cell death protein-1 (PD-1), programmed death-ligand 1 (PD-L1) and lymphocyte activation gene-3 (LAG-3) are attractive targets in immunotherapy, as they play an important role in various malignancies where they can attenuate the host immune response to tumor cells. Indeed, the PD-1/PD-L1 axis inhibits T-cell activation, proliferation, and survival [24, 25]; LAG-3 is another regulatory protein, that binds to the major histocompatibility complex class II expressed by antigen-presenting cells, competes with CD4 and triggers inhibitory signaling that suppresses T-cell function [26, 27].

New interesting combinatorial strategies were recently used in order to fight potential escape mechanisms and increase the efficacy of BiTEs based on combinations of BiTEs with immunomodulatory monoclonal antibodies (mAbs) [28]. Examples of some of the currently ongoing clinical trials are those of Nivolumab in combination with Blinatumomab compared to Blinatumomab alone for patients with B-cell Acute Lymphoblastic Leukemia (*NCT04546399*); phase I/II trial of pembrolizumab and Blinatumomab for recurrent or refractory Acute Lymphoblastic Leukemia (*NCT03512405*), phase Ib trial of Cibisatamab in combination with atezolizumab in patients with locally advanced and/or Metastatic CEA-Positive Solid Tumors (*NCT02650713*).

A further progress we identified was the generation of multi-specific T-cell engagers targeting also the ICs. Recently, novel generation of bi-specifics targeting two distinct ICs on T cells to overcome resistance of solid tumors due to checkpoint inhibition has led to the approval of the dual PD-1/CTLA-4 checkpoint inhibitory bi-specific, called Cadonilimab, for treatment of patients with relapsed or metastatic cervical cancer and to a large number of ongoing clinical trials with others such as those combining PD-L1 and CTLA-4 or Tim3 targeting moieties [29].

In our laboratory, as a further progress in the field, we generated a series of multi-specific tribodies, called 53X tribodies, which represent an innovative approach, since they are, for the first time, tri-specific constructs, made up of a Fab arm binding to the TAA, called 5T4, and two different scFvs, the first binding to CD3, and a second scFv derived from an antibody specific for one of the following ICs: PD-1 (in 53D), PD-L1 (in 53L10), or LAG-3 (in 53 G), as previously described [30]. The idea was to combine the efficacy of T-cell engagement, through an artificial synapse, of the cancer cells expressing 5T4 with modulation of the ICs pathways in order to overcome immunosuppression exerted by a solid tumor environment [30].

5T4 is an oncofetal antigen, which plays a major role in cancer progression as it can promote tumor cell migration and survival and is overexpressed in many types of cancers, such as colorectal, ovarian, breast, lung, and renal cancers, but rarely expressed or absent in healthy adult tissues [31, 32].

In this study, we compare the biological properties of the novel 53X tribodies to those of the corresponding immunomodulatory mAbs, currently in clinical use, targeting the same ICs, such as relatlimab (anti-LAG-3), atezolizumab (anti-PD-L1) and pembrolizumab (anti-PD-1) [33, 34], either used alone or in combination with a control bi-specific tribody, called 53 P, targeting 5T4 but devoid of anti-IC scFvs.

## Results

### Binding of tri-specific tribodies to their targets purified or expressed on cells, in comparison with the clinically validated mAbs

**Fig. 1:**
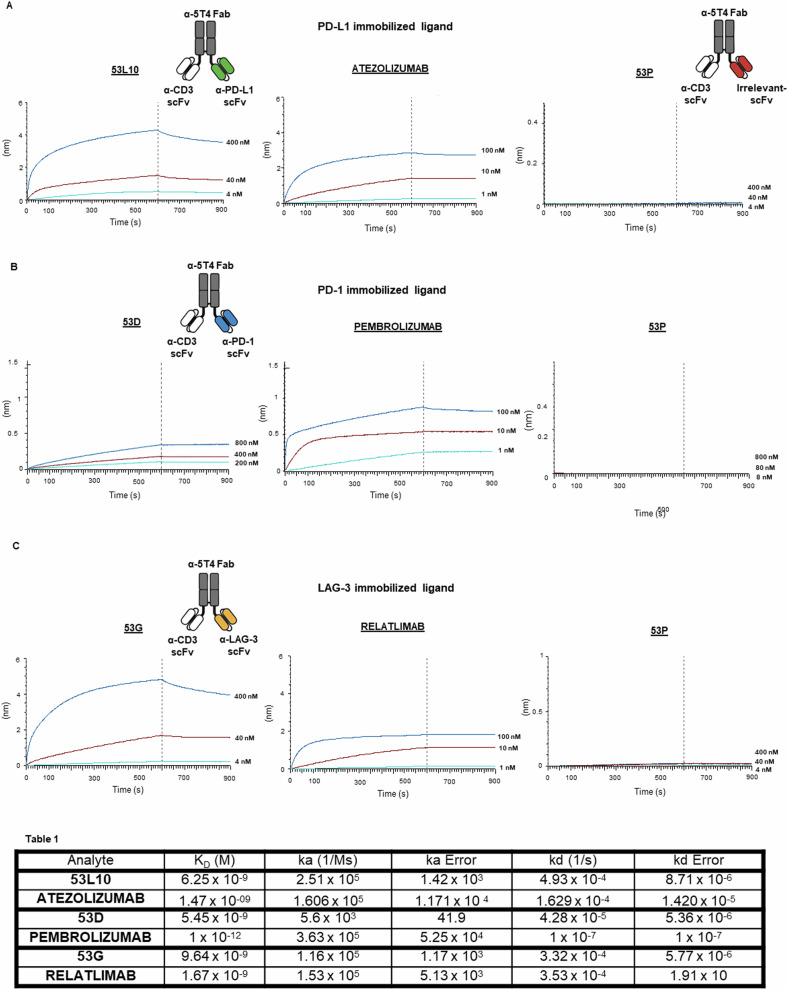
Schematical representation of 53X tribodies and their binding kinetics, compared to clinically validated mAbs, on their corresponding purified targets via BLI analyses. The sensorgrams reported in **A** for 53L10 compared to atezolizumab, **B** for 53D compared to pembrolizumab, and **C** for 53 G compared to relatlimab were obtained via BLI analyses. The recombinant PD-L1/Fc, PD-1/Fc, and LAG-3/Fc were used as ligands, whereas the tri-specific tribodies or validated mAbs were used as analytes and tested at increasing concentrations. As a negative control, the tribody 53 P was used as an analyte in a parallel assay. The sensorgrams show the association and dissociation rates of the analytes. The schematical representations of 53X tribodies are reported as insets in each relative binding panel. The constructs contain a Fab domain specific for 5T4 and an anti-CD3 scFv fragment; the additional scFv fragment targets an IC, i.e., PD-L1 (**A**), PD-1 (**B**), or LAG-3 (**C**), respectively. The scFv from Palivizumab was used for 53 P tribody, generated as a control (**A**). *K*_D_ values and association and dissociation constants of the binding of each analyte to its corresponding immobilized ligand on a pro-A biosensor, processed according to the following formula: in which A represents the analyte, and B represents the immobilized ligand. Ka and kd are the association and dissociation rate constants [50]. Analysis was performed using Octet Analysis Studio 13.0 Software (Sartorius, Fremont, CA, USA).

The novel tribodies, called 53L10, 53D, and 53 G were generated, as previously reported [30], by our research team by fusing a Fab derived from an anti-5T4 mAb and two scFvs derived from an anti-CD3 mAb, and an anti-IC mAb (anti-PD-L1, anti-PD-1, or anti-LAG-3 mAb) respectively, see insets of Fig. 1. These novel constructs were found to have more potent anti-tumor activity than parental monospecific or T-cell engager bi-specific mAbs, since they combine the T-cell recruiting ability of the parental bi-specific anti-5T4/CD3 with the immunomodulatory function of the anti-IC antibodies [30].

Here, we decided to compare their properties to those of immunomodulatory antibodies currently in clinical use and targeting the same ICs, i.e. the anti-LAG-3 (relatlimab), anti-PD-L1 (atezolizumab), and anti-PD-1 mAbs (pembrolizumab) [33, 34].

In parallel assays, a negative control tribody containing an irrelevant-scFv, called 53 P, derived from the same parental anti-5T4/CD3 tribody but lacking the immunomodulatory antibody fragment and including an unrelated scFv derived from the anti-respiratory syncytial virus mAb (Palivizumab) [35], was used.

We first tested their binding to recombinant ICs immobilized on a biosensor by biolayer interferometry (BLI). As shown in Fig. 1, 53 P, as expected for the negative control, did not show any significant binding to the immobilized PD-L1 (Fig. 1A), PD-1 (Fig. 1B), or LAG-3 (Fig. 1C), whereas 53L10, 53D and 53 G bind to their corresponding purified targets with a satisfactory *K*_D_ of 5–7 nM, as reported in Fig. 1. The clinically validated mAbs show even lower *K*_D_, likely due to their bivalent binding, compared to the monovalent binding of immunomodulatory scFvs inserted in the tribodies. The tribody 53D showed the lowest affinity for the IC target (PD-1) and was tested at higher concentrations to measure the affinity.

**Fig. 2:**
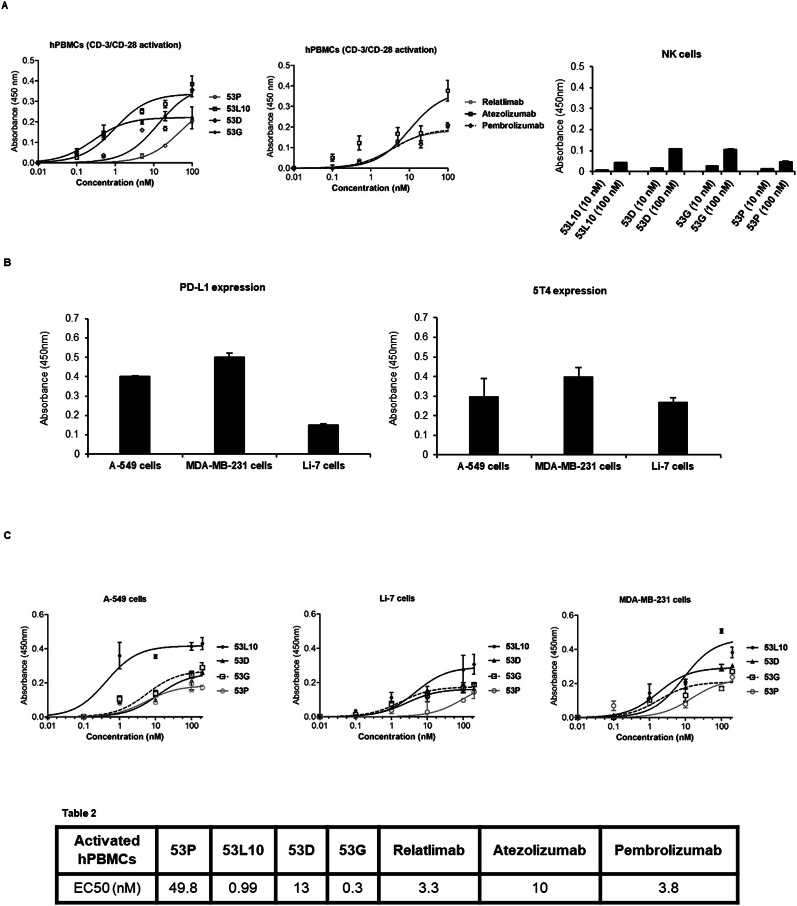
Binding curves of 53X tribodies on lymphocytes and tumor cells tested in comparison with the clinically approved mAbs. **A** The tribodies and the FDA approved mAbs were tested at the indicated concentrations in parallel assays by cell ELISA on human lymphocytes activated with anti-CD3/CD28 beads. As a negative control, the binding of the novel tribodies was tested also on natural killer cells stimulated with SEB (50 ng/ml) for 72 h. **B** Expression of 5T4 and PD-L1 on a panel of tumor cells. Cell ELISA assays to check the expression level of 5T4 and PD-L1 on lung A-549, breast MDA- MB-231 and liver Li-7 cancer cells were performed with a commercial anti-PD-L1 or anti-5T4 antibody. **C** Binding curves of 53X tribodies tested at increasing concentrations (0.1–100 nM) on the indicated tumor cell lines. Binding values were reported as the mean of determinations obtained in three independent experiments. Error bars depicted means ± SD. The table reports the EC50 values obtained by ELISA binding curve analyses with Prism (Graphpad) tool according to the following model: *Y*  =  Bmax*X/(Kd+*X*)  +  NS*X + Background. EC50 values are obtained by ELISA binding curve analyses with Prism (Graphpad) tool according to the following model: *Y*  =  Bmax*X/(Kd+ *X*)  +  NS*X + Background.

Even though 53 G and 53L10 show lower affinity for the recombinant proteins compared to relatlimab and atezolizumab, they show comparable or even higher binding affinity to the activated lymphocytes likely due to the inclusion of the anti-CD3 moiety in the tribodies. Indeed, the apparent affinity of 53L10 and 53 G, when tested by cell enzyme-linked immunosorbent assays (ELISA) on activated lymphocytes (Fig. 2A) are in the subnanomolar range (0.2–0.9 nM) compared to those of atezolizumab and relatlimab in the nM range. 53D instead shows a higher EC50 of 13 nM compared to that of pembrolizumab of 3 nM in line with its lower binding affinity observed also in previous BLI analyses.

We then decided to test their effects on tumor cells. To this aim, we first examined the expression levels of 5T4 and PD-L1 on the surface of different tumor cell lines by cell ELISA. As shown in Fig. 2B, the 5T4 expression was detectable at sufficient (reliable absorbance values ≥ 0.1) levels on the surface of A-549, MDA-MB-231, and Li-7 cell lines derived from lung, breast cancer, and hepatocellular carcinoma, respectively, whereas PD-L1 was expressed at higher levels on A-549 and MDA-MB-231 than Li-7 cells.

Thus, the novel tribodies were tested on these cell lines for their binding to the tumor cell surface by cell ELISA. In parallel assays, an unrelated IgG was used as a negative control. As shown in Fig. 2C, all the tribodies showed the ability to bind to tumor cells in nM range, whereas as expected, the unrelated IgG did not show significant binding (data not shown). In particular, 53L10 reached the highest binding affinity likely due to the dual binding ability to both 5T4 and PD-L1.

This hypothesis is confirmed by its lower binding to Li-7 cells, which express lower levels of PD-L1, compared to that observed on the other two cell lines expressing higher levels of PD-L1.

As a negative control, we also analyzed the binding of the novel tribodies on NK cells that express low levels of PD-1, PD-L1, and LAG-3, and do not have 5T4 or CD3 [36]. To this aim, NK cells were isolated from human peripheral blood mononuclear cells (hPBMCs) by using a kit (MACS, Miltenyi Biotec, Bergisch Gladbach, CO, Germany), activated with SEB for 48 h, and used for cell ELISA with the tri-specific tribodies, used at concentrations of 10 and 100 nM. As expected the binding was significantly reduced for all the tribodies compared to those observed either on tumor cells or activated hPBMCs (see Fig. 2A).

### Comparison of cytotoxic activity of novel immunomodulatory tribodies with that of 53 P control tribody in combination with the validated mAbs

**Fig. 3:**
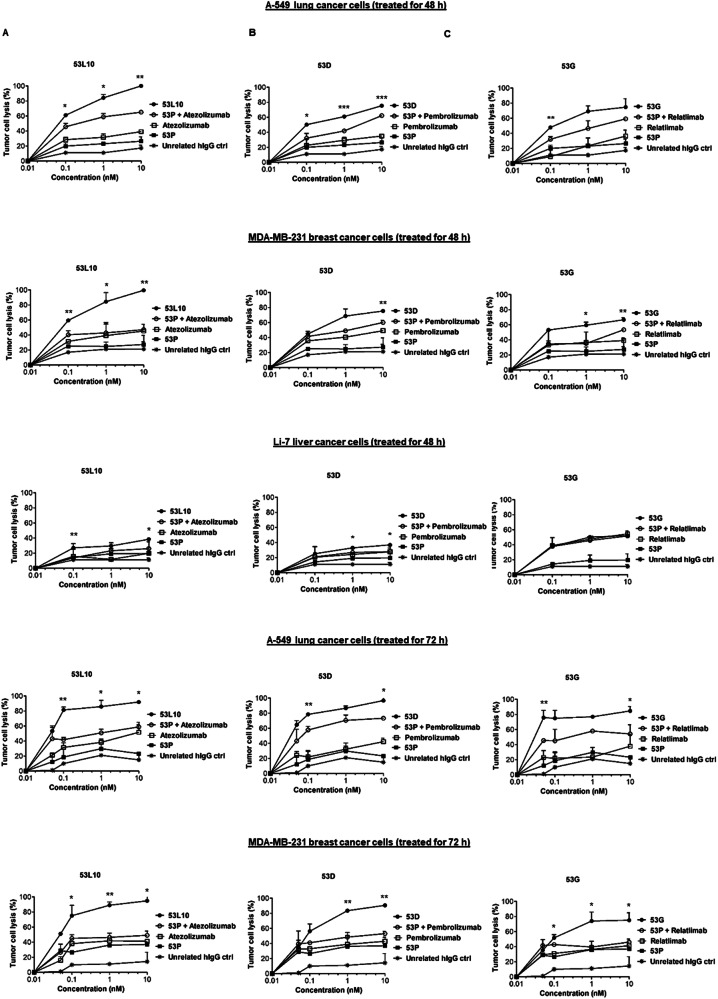
Cytotoxic effects of the novel tribodies in comparison with the combinations of 53 P and the clinically validated mAbs. Tumor cells (A-549, MDA-MB-231, and Li-7) were co-cultured with hPBMCs (3:1 effector/target ratio) and treated for 48 h or 72 h with 53 P (■), 53L10 (**A**), 53D (**B**), and 53 G (**C**) tribodies (all ●). The clinically validated immunomodulatory mAbs atezolizumab, pembrolizumab, and relatlimab (all □), or their combinations with 53 P (**○**) were tested in parallel at the indicated concentrations (0.05–10 nM). Co-cultures untreated or treated with an unrelated IgG (*) were used as negative controls. Cell lysis was measured by detecting LDH release. Error bars depicted means ± SD. *P* values were calculated by comparing each treatment with the tri-specific tribody to that of the corresponding combinatorial treatment (53 P + mAb targeting the same IC), and the *P* values reported are: ***P ≤ 0,001; **P < 0.01; *P < 0.05, by student’s *t* test (two variables).

To compare the efficacy of the novel tribodies targeting PD-L1, PD-1, and LAG-3 with that of clinically validated mAbs targeting the same ICs combined with the control tribody lacking the immunomodulatory moiety, their effects were investigated in a co-culture system. To this aim, MDA-MB-231, a human breast cancer cell line, and A-549, a human alveolar basal epithelial adenocarcinoma cell line, that express higher levels of PD-L1 and 5T4, were co-cultured with hPBMCs (3:1 effector/target ratio) and incubated at 37 °C for 48 h or 72 h in the absence or presence of 53D, 53 G, and 53L10. In parallel assays, the negative control tribody, containing an irrelevant-scFv (53 P), derived from the same parental tribody but lacking the immunomodulatory antibody fragment, was used alone or combined with the validated anti-PD-1 (pembrolizumab), anti-PD-L1 (atezolizumab) or anti-LAG-3 (relatlimab) mAb. After the treatments, cytolysis was measured by lactate dehydrogenase (LDH) released into the cell supernatant using LDH assay kit. The results showed that all novel tribodies, with the exception of 53 P, used as a negative control, induced tumor cytotoxicity with greater efficacy than the combination of each mAb in clinical use with 53 P (see Fig. 3). In particular, the tribody 53L10 induced a two-fold increase of LDH release compared to the combinations of the corresponding clinically validated mAbs and 53 P control. As a control for specificity, Li-7 cancer cells expressing lower levels of PD-L1 and 5T4 (Fig. 3) compared with the above-mentioned cell lines, were used in parallel assays and the tribodies showed lower increases of cytotoxic effects than their corresponding combinations, in comparison with the two highly PD-L1-positive cell lines mentioned above, thus confirming the specificity of the treatments. When the treatments on the latter two cell lines were prolonged to 72 h, the stronger cytotoxic effects of the novel tribodies were observed even at the lower dose tested (Fig. 3).

**Fig. 4:**
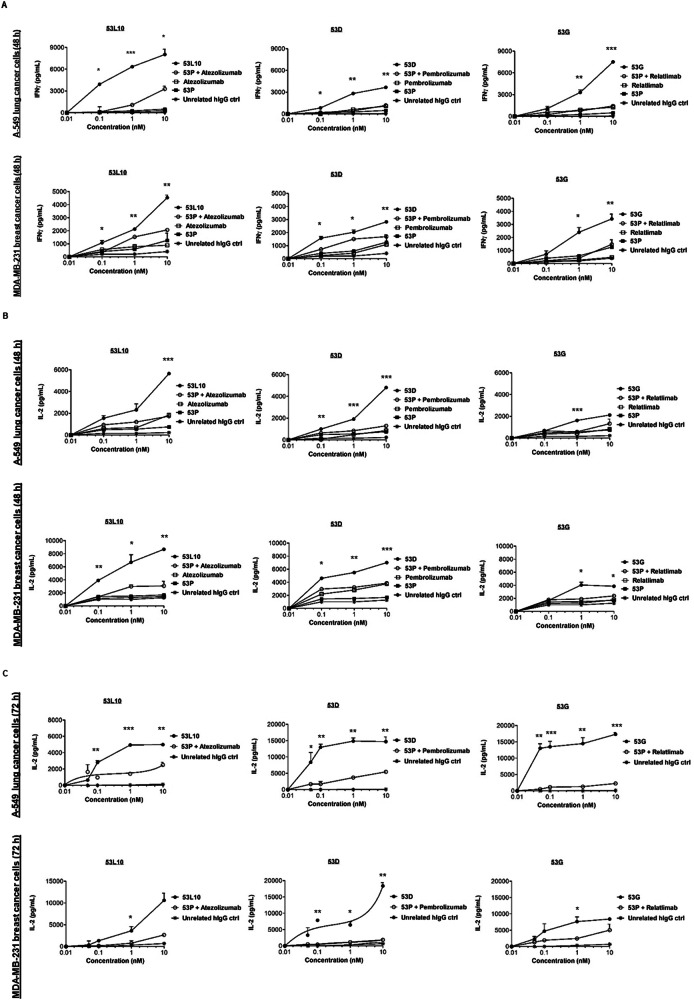
IFNγ and IL-2 release in co-cultures of tumor cells with hPBMCs treated with the novel tribodies. Tumor cells (A-549 and MDA-MB-231) were co-cultured with hPBMCs (3:1 effector/target ratio) and treated for 48 h or 72 h with 53 P (■), 53L10, 53D and 53 G tribodies (●). The parental immunomodulatory mAbs atezolizumab, pembrolizumab, and relatlimab (□), or their combinations with 53 P (**○**) were tested in parallel assays, for comparison, at the indicated concentrations (0.05–10 nM). The levels of IFNγ (**A**) and IL-2 (**B**, **C**) released in the supernatant of co-cultured cells were measured by cytokine secretion kit (R&D systems), following the manufacturer’s recommendations. Co-cultures untreated or treated with an unrelated IgG (*) were used as negative controls. Error bars depicted means ± SD. *P* values were calculated by comparing each treatment with the tri-specific tribody to that of the corresponding combinatorial treatment (53 P + mAb), and the *P* values reported are: ***P ≤ 0.001; **P < 0.01; *P < 0.05, by student’s *t* test (two variables).

To clarify whether the enhancement of tumor cell lysis by the novel tribodies was combined with increased secretion of cytokines by lymphocytes, the concentration of interferon γ (IFNγ) and interleukin-2 (IL-2), functional markers for hPBMC activation [37, 38], released in the culture supernatants of those co-cultures was measured, and the effect of the novel tribodies was compared with the effect of 53 P in combination with each of the mAbs in clinical use. As shown in Fig. 4A, B, levels of IFNγ and IL-2 for cells treated for 48 h, and in Fig. 4C for those treated for 72 h (IL-2), were much higher in the supernatants of the co-cultures of tumor cells and hPBMCs treated with all the novel tribodies compared to those observed in the combinatorial treatments of their 53 P control tribody with pembrolizumab, atezolizumab or relatlimab, respectively. The tribodies induced the release of IL-2 and IFNγ in a dose-dependent manner, by increasing 4- or 5-fold the levels of cytokines compared to the combinatorial treatments.

To better verify whether the increased secretion of cytokines was indeed due to specific activation of T cells, we also performed staining of T-cell activation markers. To this aim, A-549 and MDA-MB-231 cells were plated for 4 h, then hPBMCs were added in the absence or presence of 53L10, 53 P, atezolizumab, and 53 P in combination with atezolizumab, used at a concentration range of 0.1–10 nM. The mixtures were then incubated at 37 °C for 24 h or 48 h.

**Fig. 5:**
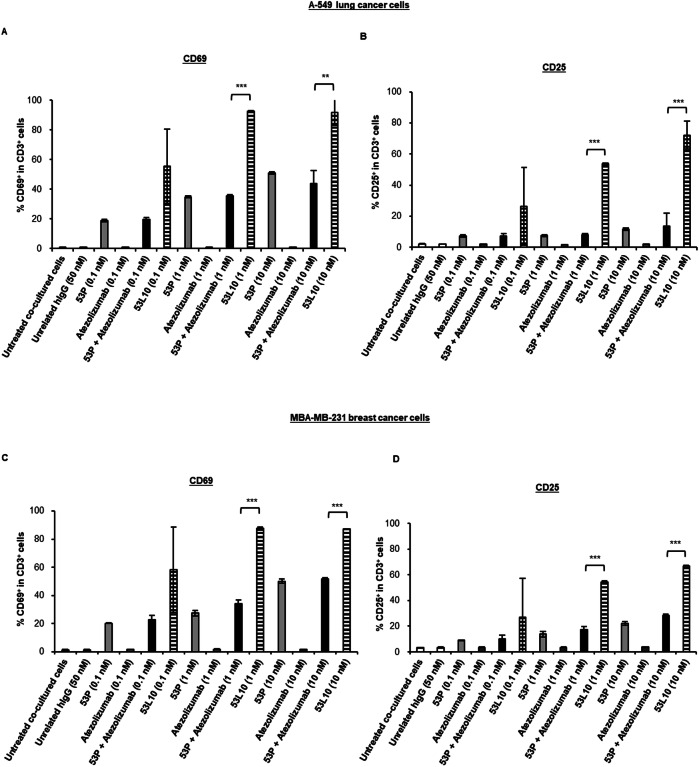
T-cell activation by 53L10 tribody in comparison with the combination of 53 P and atezolizumab. Tumor cells (A-549 and MDA-MB-231) were co-cultured with commercially available hPBMCs (10:1 effector: target ratio) and treated with 53 P (light gray), 53L10 (striped), atezolizumab (dark gray) and 53 P plus atezolizumab (black) at the indicated concentrations for 24 h and 48 h. As negative controls, both untreated cells or treated with an unrelated IgG (empty bars) were used. T-cell activation was directly measured by percentage of CD69-positive cell population in CD3-positive cells at 24 h and percentage of the CD25-positive cell population in CD3-positive cells at 48 h by flow cytometry. **A**, **C** Percentage of CD69^+^ population in CD3^+^ hPBMCs co-cultured with A-549 (**A**) or MDA-MB-231 cells (**C**). **B**, **D** Percentage of CD25^+^ population in CD3^+^ hPBMCs co-cultured with A-549 (**B**) or MDA-MB-231 cells (**D**). The data were expressed as mean ± SD. Statistical significance was calculated by student’s *t* test (two variables). *P* values reported are: ***P ≤ 0,001; **P < 0.01.

After 24 h of treatment, the cells were stained with the FITC anti-human CD3 antibody and APC anti-human CD69 antibody, whereas after 48 h of incubation they were stained with the FITC anti-human CD3 antibody and APC anti-human CD25 antibody. The expression analyses of both the markers, measured by flow cytometry, demonstrated that 53L10 induced more significant staining of T-cell activation markers than 53 P, atezolizumab, or their combination (see Fig. 5).

In particular, as shown in Fig. 5, in both co-cultures of A-549 (Fig. 5A, B) or MDA-MB-231 (Fig. 5C, D) with hPBMC, 53L10 induced up-regulation of CD69 at 24 h and CD25 at 48 h in a dose-dependent manner. Interestingly, 53L10 alone induced even stronger T-cell activation than the combination of 53 P and atezolizumab. These results suggest that 53L10 can efficiently activate T cells directly, resulting in increased secretion of cytokines such as IFNγ and IL-2. It has been reported in literature that increased levels of PD-L1 expressed on the surface of tumor cells, in specimens that are freshly isolated from patients with cancer, were found to be induced by IFNγ [39]. Indeed, PD-L1 is mainly regulated by the type II interferon receptor signaling pathway through JAK1, JAK2, and STATs, leading to the binding of IRF1 with the PD-L1 promoter. Thus we decided to analyze the effects of IFNγ at the concentrations detected in these co-cultures (about 10 ng/mL) treated with TRBs on PD-L1 levels of co-cultured tumor cells.

To this aim, the tumor cells were incubated for 48 h with IFNγ at the concentration of 10 ng/mL, and then they were used for cell ELISA assays in comparison with the untreated cells, to detect the levels of PD-L1 by using commercial anti-PD-L1 Ab. In parallel assays, we also analyzed the levels of 5T4 before and after the treatment with the anti-5T4 bivalent Tb535 [30]. We found that, indeed, the level of PD-L1 significantly increased especially in Li-7 and A-549 cells (Supplementary Fig. 1), whereas the level of 5T4 was not significantly affected, as expected. Therefore, tumor environment and treatment with T-cell engagers both induce PD-L1 expression with a possible negative feedback mechanism, thus allowing the tumor cells to escape immune system surveillance. Thus, the insertion of the anti-PD-L1 scFv in 53L10 can lead not only to higher binding ability to tumor cells, but can also significantly potentiate the activation of T cells and consequent tumor cell killing by efficiently interfering with the increased PD-L1-PD-1 interaction (see Supplementary Fig. 2).

Thus, these results clearly confirm the validity of the strategy to insert an immunomodulatory moiety in the bi-specific tribody to increase its anti-tumor efficacy and importantly, indicate that the resulting immunomodulatory tri-specific tribody is more effective than the combination of 53 P with the clinically used anti-IC antibody.

### Comparison of cytotoxic activity of novel immunomodulatory tribodies with that of 53 P control tribody in combination with immunomodulatory Bi-specifics

To further analyze the advantages of the novel 53X tri-specific tribodies over the bi-specifics or their combinations, we also compared the effects of 53L10, 53D, and 53 G to those of control 53 P (5T4/CD3/unrelated) combined with immunomodulatory bivalent bi-specific tribodies, called 0304 (PD-L1/LAG-3) and 0506 (PD-1/LAG-3), previously generated in our laboratory by fusing the anti-PD-L1 or anti-LAG-3 Fab with the anti-PD-1 or anti-LAG-3 scFvs (see Supplementary Fig. 3) [40].

**Fig. 6:**
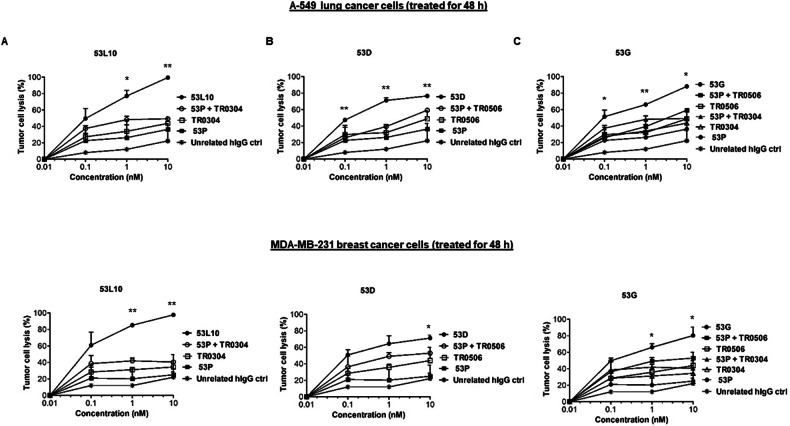
Comparison of cytotoxic effects of 53X tribodies on tumor cells with the combinations of bi-specifics and 53 P. Tumor cells were co-cultured with hPBMCs (3:1 effector/target ratio) and treated for 48 h with 53 P (■), 53L10 (**A**), 53D (**B**) and 53 G (**C**) tribodies (●). The bi-specific immunomodulatory mAbs TR0304 and TR0506 (□), or their combinations with 53 P (**○**) were tested in parallel assays at the indicated concentrations (0.1–10 nM). Co-cultures untreated or treated with an unrelated IgG (*) were used as negative controls. Cell lysis was measured by detecting LDH release. Error bars depicted means ± SD. *P* values were calculated by comparing each treatment with the tri-specific tribody to that of the combination of 53 P with the bi-specific tribodies containing the same targeting domain, and the *P* values reported are: **P < 0.01; *P < 0.05, by student’s *t* test (two variables).

To this aim, we incubated MDA-MB-231 and A-549 cell lines, that express higher levels of PD-L1 and 5T4, with hPBMCs and incubated at 37 °C for 48 h in the absence or presence of 53D, 53 G, and 53L10 (3:1 effector/target ratio). In parallel assays, the control tribody (53 P) derived from the same parental tribody but lacking the immunomodulatory antibody fragment, was used alone or combined with the bi-specific immunomodulatory tribodies 0304 and 0506. After the treatments, tumor cell lysis was measured by measuring LDH released into the cell supernatant. The results showed that all novel tribodies induced a more potent tumor cytotoxicity than the combination of 53 P with each immunomodulatory bi-specific targeting PD-L1 or PD-1 and LAG-3 (see Fig. 6).

### In vivo effects of 53L10 in comparison with atezolizumab and the control 53 P tribody

**Fig. 7:**
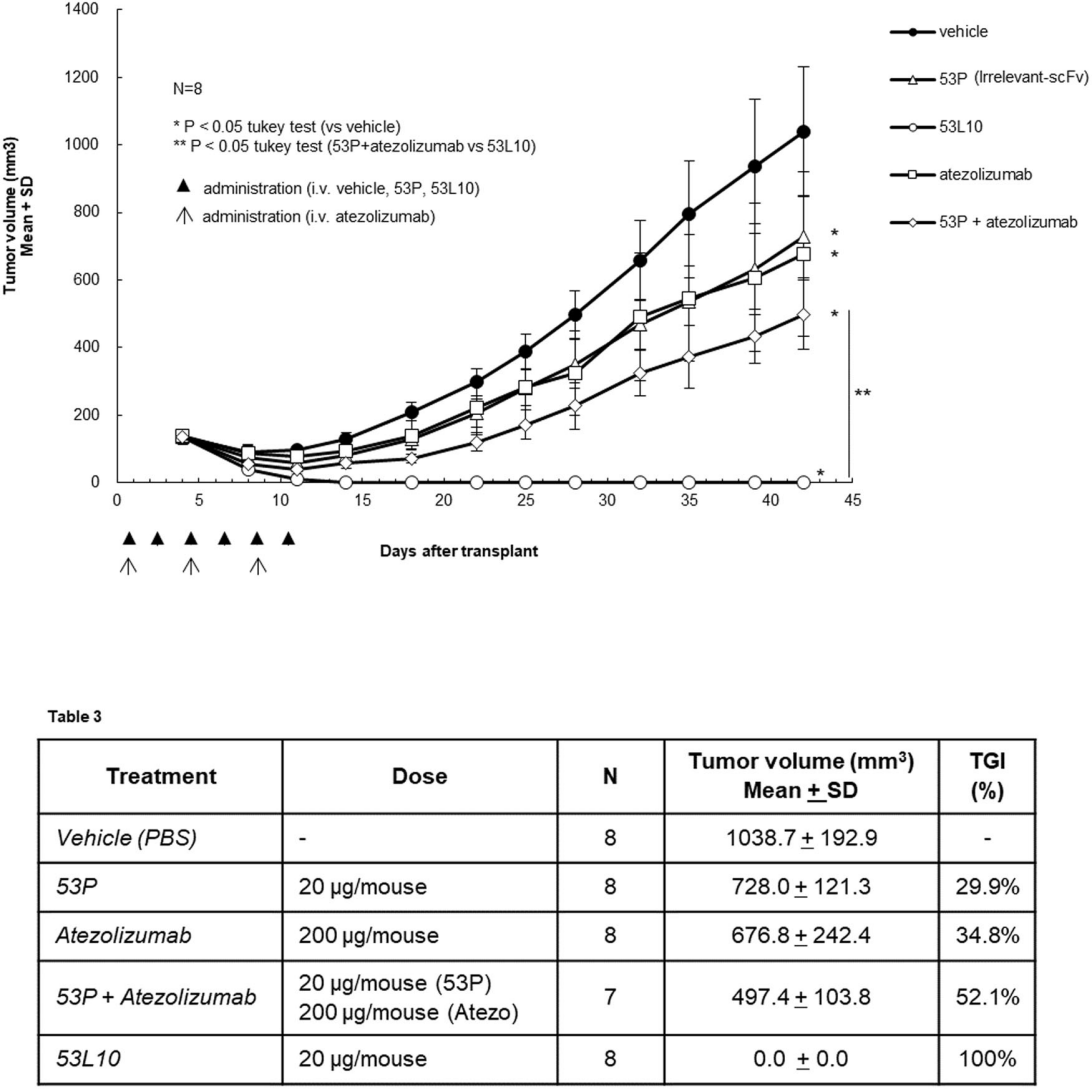
In vivo anti-tumor efficacy of the novel tribody 53L10 compared with atezolizumab, 53 P and their combination. Tumor growth curves of A-549 subcutaneous tumors treated with atezolizumab or the novel tribody 53L10 in the presence of human PBMCs. In particular, 5 × 10^6^ A-549 cells mixed with the same number of activated human PBMCs were subcutaneously transplanted into the right flank of NOD/SCID mice. hPBMCs were activated by stimulation with Dynabeads Human T-Activator CD3/CD28 (Veritas) for 4 days before transplantation and then injected. Intravenous administrations of vehicle (PBS; ●), 53 P (△), or 53L10 (○) on Days 0, 2, 4, 6, 8, and 10 (6 times in total) after cell transplantation were performed at the doses indicated in the figure. Intravenous administrations of atezolizumab (□) at the dose of 200 μg were performed on Days 0, 4, and 8 (3 times in total as indicated by the arrows). The combined treatment of 53 P with atezolizumab (◇) was carried out by administering 53 P on Days 0, 2, 4, 6, 8, 10 and atezolizumab on Days 0, 4, 8. Tumor volumes are expressed as means ± standard deviation (SD). Tukey’s multiple test (for all the combinations) was performed as a significance test. *P < 0.05 vs vehicle (PBS) group, **P < 0.05 (53 P + atezolizumab vs 53L10). The table reports the tumor volume and TGI in each treatment group at Day 42 (final day of the study). Three tumor volume and TGI obtained in each treatment group on Day 42 (final day of the in vivo study).

The in vivo anti-tumor efficacy of 53L10, 53 P, and atezolizumab (the clinically validated anti-human PD-L1 antibody, which showed in vitro the highest efficacy among the validated mAbs) was compared in A-549 (human alveolar basal epithelial adenocarcinoma cell line) xenograft mouse model (Fig. 7). A cell suspension containing 5 × 10^6^ A-549 cells mixed with the same number of activated human PBMCs was subcutaneously transplanted into the right flank of NOD/SCID mice (Day 0). From the day of transplantation, administration of vehicle (PBS), 53 P (20 μg/mouse), or 53L10 (20 μg/mouse) was performed every other day for a total of six times (Days 0, 2, 4, 6, 8, and 10). Intravenous administration of atezolizumab (200 μg/mouse) alone or in combination with 53 P was performed 3 times in total on Day 0, Day 4, and Day 8.

Tumor volume was measured 4 days after transplantation and thereafter twice a week until 42 days after transplantation when tumor volume in the vehicle treatment group reached 1000 mm^3^ (see Fig. 7).

In the 53L10 treatment group (20 μg/mouse), the highest tumor growth inhibition effect (TGI of 96–100%) was observed with complete tumor regression in 5 out of 8 individuals on the final day of the test (day 42). Significant but (35% TGI) much lower anti-tumor efficacy was observed in the atezolizumab (200 μg/mouse) treatment group compared with the vehicle (PBS) treatment group (Fig. 7).

The control 53 P tribody showed slight anti-tumor efficacy comparable to that previously reported [30], reaching a maximum of about 30% growth inhibition. The combined treatment of atezolizumab and 53 P showed a more potent effect than single-agent treatments (about 50% TGI) but was unable to induce the tumor regression reached by 53L10.

## Discussion

Bi-specific antibodies (BsAbs) have become significantly more interesting and succesful over conventional mAbs in cancer immunotherapy in the past few years. Three BsAbs have been approved by FDA within 2014, and in the last 3 years additional 11 bsAbs received FDA approval [29, 41].

In particular, the T-cell engaging BsAbs simultaneously binding with one arm to a TAA on cancer cells and with the other one to CD3 complex on T cells to form a TCR-independent artificial immune synapse, can represent a valuable and safe alternative strategy to CAR-T cells due to their lower cost and complexity of clinical management, the lack of unsafe viral vectors as well as the advantage of better tuning and monitoring of eventual side effects, such as cytokine storm and systemic T-cell activation, by easy adjustments of therapeutic doses or discontinuation of the treatment that cannot be easily obtained with CAR-T-cell treatment [6–8].

Currently, more than 100 BsAbs are in clinical trials, and demands to develop new and innovative therapeutic BsAbs are increasingly growing [41]. In this regard, we designed and produced a novel generation of tri-specific tribodies that not only can bind to 5T4 antigen on tumor cells and TCR/CD3 complex on the cytotoxic T cells to activate T cells bypassing TCR/MHC recognition, but they could also be resistant to cancer immunosuppressive environment. Indeed, they are made up of the anti-5T4 Fab, the CD3 binding scFv, and an additional scFv derived from an antibody targeting a specific IC. In particular, 53L10 tribody includes the scFv specific for PD-L1; the 53D and 53 G those specific for PD-1 and for LAG-3, respectively [30].

Here, we tested their binding to purified targets, tumor cells, and lymphocytes and their functional properties on tumor cells in comparison with the clinically validated immunomodulatory mAbs targeting the same ICs, i.e. the anti-LAG-3 (relatlimab), anti-PD-L1 (atezolizumab), and anti-PD-1 mAbs (pembrolizumab), used alone or in combination with a control bi-specific tribody devoid of the immunomodulatory scFv, which contains instead an unrelated scFv derived from the anti-viral Palivizumab antibody (called 53 P).

We found that the novel tribodies were able to activate hPBMCs more efficiently than clinically validated mAbs either used alone or in combination with 53 P in co-culture-based assays, leading to a stronger tumor cytotoxicity and cytokines release. In particular, one of them, called 53L10, a tri-specific T-cell engager targeting 5T4 and PD-L1, showed the most promising anti-tumor efficacy in vitro by inducing 80–100% of tumor lysis at very low concentrations. These results were further analyzed by measuring the secretion in the supernatant of cytokines (IL-2 and IFNγ) by ELISA assays and up-regulation of CD69 and CD25 in treated hPBMCs by FACS. In both cases, 53L10 activated T cells more efficiently than 53 P, atezolizumab, or their combination. Furthermore, 53L10 led to complete tumor regression in vivo, showing much higher anti-tumor efficacy than atezolizumab (used at tenfold higher concentration), 53 P, and their combination, which reached a maximum of 50% TGI.

We have also shed light on its mechanism of action as we found that the treatment with the bi-specific tribody induces activation of T cells against cancer cells and ultimately leads to IFNγ secretion, which in turn can induce the expression of PD-L1 on tumor cells with a possible negative feedback mechanism, thus allowing the tumor cells to escape the immune system. Therefore, the insertion of the anti-PD-L1 scFv in the construct can lead not only to the higher binding ability of the tri-specific antibody to tumor cells but can also significantly potentiate the activation of T cells and consequent tumor cell killing by efficiently interfering with the increased PD-L1-PD-1 interaction.

Instead when the two immunoagents are administered separately, the bi-specific construct binds to tumor cells with one arm and T cells with the other arm whereas the anti-IC mAb could bind to other cell populations such as tumor-associated macrophages, fibroblasts, mast cells, B cells, and T cells within solid tumors that may become PD-L1^+^ in response to IFNγ. This could well explain the more potent anti-tumor efficacy of the tri-specific tribody compared to the combination of conventional T-cell engager (53 P) and the immunomodulatory mAbs. Furthermore, this clarification could be of critical importance also for the design of novel T-cell engager constructs targeting other tumor antigens.

These tri-specific constructs could represent innovative tools for cancer therapies as no similar constructs have been yet approved by FDA for clinical use, even though some are in clinical trials with the format TAA/CD3/CD28 [11, 42]. However, in comparison with 53L10, they contain anti-CD28 binding domains that in the past have been associated with the risk of severe side effects due to excessive cytokine release that could be overcome by the use of anti-PD-L1 strategy, which is mainly effective on exhausted T cells. On the other side, in the last 3 years 11 bsAbs received FDA approval, with nine of them in oncology and 7 T-cell engagers, thus showing the potential of this novel class of T-cell engager therapeutics [41].

Thus, altogether these results clearly confirm the validity of the strategy of inserting an immunomodulatory moiety in the bi-specific tribody to increase its anti-tumor efficacy and open up the possibility to extend this strategy to many other T-cell engagers constructs targeting a large variety of TAA even though further studies in more sophisticated in vivo models, such as humanized mouse models, by using the novel tribodies or the combinations with validated mAbs should be considered in the future due to the lack of significant binding to mouse targets of some immunoagents.

## Materials and methods

### Antibodies and human recombinant proteins

The human commercial antibodies used are reported below:

PD-L1/B7-H1 polyclonal antibody (AF156, R&D Systems, Minneapolis, MN, USA); CD223 (anti-LAG-3) polyclonal antibody (PA5-97917, Invitrogen, Rockford, IL, USA); anti-human IgG (Fab’)2 goat HRP-conjugated mAb (ab87422, Abcam, Biomedical Campus, Cambridge, UK) and HRP-conjugated 6*His, His-Tag mAb (HRP-66005, Proteintech, Rosemont, IL, USA).

The clinically validated antibodies used are the following:

anti-PD-L1 atezolizumab mAb (N298A, InvivoGen, San Diego, CA, USA); anti-PD-1 pembrolizumab (L01FF02, Merck Sharp & Dohme B.V., Haarlem, The Netherlands); anti-LAG-3 relatlimab (BMS-986016, MedChemExpress, DBA, Segrate, MI, Italy);

The following human recombinant proteins were used:

LAG-3 Fc Chimera Protein (2319-L3), PD-L1/B7-H1 Fc Chimera Protein (156-B7), PD-1 Fc Chimera Protein (1086-BD) and IgG1 Fc Protein (110-HG) (all from R&D Systems, Minneapolis, MN, USA).

### Tumor cell lines

The tumor cell lines used were: human triple-negative breast cancer (TNBC) MDA-MB-231 cell line and non-small-cell lung cancer A-549 cell line, obtained from the American Type Culture Collection (Rockville, MD, USA); human Hepatocellular Carcinoma (HCC) Li-7 cell line from Cytion (Tebubio, NV Duwijckstraat, Lier, Belgium).

MDA-MB-231 cells were cultured in Dulbecco’s Modified Eagle’s Medium (11965); A-549 cells were cultured in Kaign’s Modification of Ham’s F-12 Medium (F-12 K, 15172529) and Li-7 cells were cultured in Roswell Park Memorial Institute 1640 Medium (RPMI 1640, 11875101), all media were purchased from Gibco, Life Technologies, Paisley, UK. The media were supplemented with 10% (vol/vol) heat-inactivated fetal bovine serum certified, United States (FBS, 16000044), 50 U/mL penicillin, 50 μg/mL streptomycin (15070063) and 2 mM L-glutamine (A2916801), all from Gibco Life Technologies, Paisley, UK and cultured in a humidified atmosphere of 95% air and 5% CO_2_ at 37 °C.

### Isolation of human peripheral blood mononuclear cells

Human Peripheral Blood Mononuclear Cells were thawed out starting from aliquots isolated from buffy coats previously obtained from the Blood Bank of the Medical School, as previously described [30, 36, 43–46], by using the Greiner Leucosep tubes (Z642843-300EA, Sigma-Aldrich, St. Louis, MO, USA) following the manufacturer’s instructions. Cryopreserved cell vials, previously frozen in 90% FBS plus 10% dimethyl sulfoxide (D8418, Sigma-Aldrich, St. Louis, MO, USA) solution, were gently thawed by using RPMI 1640 medium supplemented with 1% L-glutamine, 1% CTLWash™ (CTLW-010, Immunospot by Cellular Technology Limited, Shaker Heights, Cleveland, OH, USA) and 100 U/mL Benzonase (9025-65-4, Merck Millipore, Darmstadt, Germany) and washed via centrifugation. The collected lymphocytes were counted by using the automatic cell counter MUSE cell analyzer (0500–3115, Merck Millipore, Darmstadt, Germany) and incubated overnight at 37 °C in R10 medium (RPMI 1640 supplemented with 10% inactivated FBS, 1% L-glutamine, 50 U/mL penicillin, 50 μg/mL streptomycin, and 1% HEPES (15630080, Gibco, Life Technologies, Paisley, UK) before their use for functional assays.

### Isolation of NK cells

NK cells were isolated from unfractionated hPBMCs by using the human NK cells isolation kit (130-0902-657, MACS, Miltenyi Biotec, Bergisch Gladbach, CO, Germany), by following the manufacturer’s guidelines, as previously described [47].

### Production and purification of the tribodies

The tribodies 53L10, 53D, 53 G and 53 P tested, were generated by using the scFvs derived from the parental immunomodulatory mAbs [44, 48], and produced as previously described [30]. Briefly, the tribodies were obtained by co-transfecting Expi293 cells with expression vectors encoding the heavy and light chains of each construct to produce heterodimers for each tribody (53L10, 53D, 53 G and 53 P). The constructs were purified from cell culture supernatants by affinity techniques on Immobilized Metal Ion Affinity Chromatography (IMAC), as previously described [30]. The tribodies were analyzed by SDS-PAGE and size-exclusion chromatography (SEC) to test the purity and stability and the Limulus Amebocyte Lysate (LAL) test was performed to measure the level of endotoxins. The tribodies were sterilized by 0.2 µm filtration, aliquoted, and stored at −80 °C until use.

### Binding kinetic analyses by BLI

To investigate the kinetics of the novel tri-specific tribodies in comparison with the clinically validated mAbs, BLI analyses were performed by using the Octet R4 Protein Analysis System (Sartorius, Fremont, CA, USA). Biosensors carrying the protein A (18-5010, Octet Pro-A Biosensors, Sartorius, Fremont, CA, USA) were used to perform the assays, by following the manufacturer’s recommendation, as previously reported [43, 49, 50].

The main steps were the following: the Pro-A biosensors’ tips were hydrated for 15 min in 200 µL of kinetic buffer (KB) 10× (0.1% BSA, 0.02% Tween, in PBS 1×). Then, they were loaded with each human LAG-3 Fc, PD-L1 Fc, or PD-1 Fc recombinant chimeric protein (from R&D Systems), used at a concentration of 2 µg/mL, for a time interval of up to 200 s. In order to saturate the biosensors, when the analytes contained Fc, a second load with the human Fc recombinant chimeric protein (from R&D Systems) was performed, to avoid the non-specific binding of the mAbs to the pro-A biosensors. After washing, the association step was carried out by incubating the biosensors for 600 s in a solution containing the analytes (53L10, 53 P, 53D, 53 G, atezolizumab, pembrolizumab, or relatlimab) at increasing concentrations up to saturation. After the dissociation step, performed in KB buffer 10× for 300 s, the biosensors were regenerated according to the manufacturer’s recommendations. The data were acquired and processed into the Octet Analysis Studio Software 13.0 [50].

### Enzyme-linked immunosorbent assays

#### Cell ELISA on human PBMCs

The clinically validated atezolizumab, pembrolizumab, and relatlimab, or the novel generated tribodies (53L10, 53D, 53 G, and 53 P) were tested on human-activated lymphocytes to evaluate their ability to recognize the targets when expressed on cells in their native conformation. To this aim, cell ELISA assays were performed by plating lymphocytes, previously activated with Dynabeads Human T-Activator CD3/CD28 beads for 72 h (11131D, Gibco, Life Technologies, Paisley, UK), in triplicates into Nunc round-bottom 96-well plates (268200, Thermofisher Scientific, Segrate, MI, Italy) at a density of 2 × 10^5^ cells/well and incubating them with a blocking solution (PBS/BSA 6%) for 30 min at Room Temperature (RT), as previously described [46]. After blocking, the immunoagents were added at increasing concentrations (ranging from 0.1 nM to 100 nM) in PBS/BSA 3% buffer solution for 90 min at RT by gentle shaking at 350 rpm. After three extensive washes with PBS 1× the appropriate HRP-conjugated secondary antibody was added (anti-human IgG Fab Ab for the clinically validated mAbs or the His-Tag mAb for the tribodies) for 1 h at RT. After incubation, the plates were washed, and the signals were revealed by using the 3,3’, 5,5’- Tetramethylbenzidine (TMB T8665, Sigma-Aldrich, St. Louise, MO, USA) reagent. The absorbance at 450 nm was measured by an Envision plate reader (Perkin Elmer, 2102, San Diego, CA, USA) and the binding values were reported in Prism (Graphpad) tool system for the binding curve analyses and the Kd values calculation according to the model: *Y* = Bmax*X/(Kd+*X*) + NS*X + Background, as previously described [44].

#### Cell ELISA on NK cells

As control of binding specificity, the novel generated tribodies were tested by cell ELISA also on isolated NK cells. To this aim, NK cells were stimulated by incubating them with SEB (50 ng/ml) for 72 h at 37 °C and, then plated in round-bottom wells at the density of 2 × 10^5^/well. The tribodies were added at the concentrations of 10 or 100 nM, then incubated and detected by following the protocol above mentioned for hPBMCs.

#### Analysis of targets expression and binding to tumor cells

Cell ELISA assays, to check the expression level of 5T4 and PD-L1 on lung A-549, breast MDA- MB-231, and liver Li-7 tumor cells were performed by using the anti-PD-L1 commercial Ab or the parental bivalent anti-5T4 tribody, as previously described [30]. The assays were performed, by plating tumor cells into round-bottom 96-well plates at the density of 2 x10^5^/well, blocking and incubating them with 200 nM of each primary antibody for 1 h at RT. The following washes and incubation with secondary conjugated antibodies were carried out as described above.

Cell ELISA assays were also performed to analyze the ability of the novel tribodies to bind to tumor cells. To this aim, cells were plated into round-bottom 96-well plates and, after blocking, binding curves were performed by testing the tribodies in parallel assays at increasing concentrations (0.1–200 nM). The following washes, incubation with secondary antibodies and signal detection were performed as described above.

### Cytotoxicity assays

The cytotoxic effects of the tribodies were tested in co-cultures of tumor cells with human lymphocytes in comparison with the clinically validated mAbs, used alone or in combination with the 53 P. To this aim, MDA-MB-231 (1 × 10^4^ cells/well), A-549 (1 × 10^4^ cells/well), and Li-7 (1 × 10^5^ cells/well) tumor cells were plated in 96-well flat-bottom plates and incubated overnight at 37 °C; then, the lymphocytes were added (3:1 effector/target ratio) and the co-cultures treated for 48-72 h, at the concentration of 0.05, 0.1 nM, 1 nM and 10 nM, with the tribodies 53L10, 53D, 53 G or 53 P (negative control) or relatlimab, atezolizumab, or pembrolizumab mAbs, used as single agents or in combination with 53 P used at the same concentrations. In parallel assays, co-cultures of MDA-MB-231 and A-549 cells with lymphocytes were treated for 48 h with the (5T4/CD3/unrelated) 53 P control tribody used in combination with two different immunomodulatory bi-specific tribodies, previously generated [40], TR0304 (PD-L1/LAG-3) and TR0506 (PD-1/LAG-3), at the concentrations of 0.1 nM, 1 nM and 10 nM. Untreated co-cultured cells or treated with a human-unrelated IgG were analyzed as a control.

The CyQUANT LDH cytotoxicity assay was used as a method for determining the cellular cytotoxicity. Thus, after the collection of cell supernatant, the level of LDH released by the cells was evaluated to accurately and quantitatively measure the percentage of lysis of tumor cells treated [36, 51], following the manufacturer’s recommendations (C20300, Thermofisher Scientific, Rockford, IL, USA).

### Cytokine secretion assays

The supernatants of treated co-cultures were also analyzed to evaluate the secretion of IL-2 and IFNγ. The IL-2 (DY202) and IFNγ (DY285B) DuoSet ELISA Development kits (DuoSet ELISA, R&D Systems, Minneapolis, MN, USA) were used to measure the release of cytokines from cell culture supernatants.

The assays were performed as described [30, 36, 43, 44] and the absorbance values converted in pg/mL according to the producer’s recommendations, by plotting the mean absorbance for each sample relative to the standard curve constructed within the experiment.

### Staining of T-cell activation markers

Aliquots of hPBMCs from healthy donors were purchased from Cellular Technology Limited (Cleaveland, OH, USA) and thawed out according to the manufacturer’s instructions, collected after resting overnight, and counted before use. A-549 and MDA-MB-231 cells were plated in a 96-well flat-bottom plate at a density of 2 × 10^4^ cells/well for 4 h, then hPBMCs were added at effector to target ratio 10:1 in the absence or presence of 53L10, 53 P, atezolizumab and 53 P in combination with atezolizumab added at a concentration range of 0.1–10 nM. The mixtures were then incubated at 37 °C for 24 h or 48 h.

For staining of T-cell activation markers, cells were collected from the plate after 24 h and 48 h, respectively. Cells collected after 24 h incubation were stained with the FITC anti-human CD3 antibody (Biolegend, San Diego, CA, USA) and APC anti-human CD69 antibody (Biolegend). Cells collected after 48 h of incubation were stained with the FITC anti-human CD3 antibody (Biolegend) and APC anti-human CD25 antibody (Biolegend). The expression of CD3 and CD69 after 24 h and the expression of CD3 and CD25 after 48 h were measured by flow cytometry using FACSLyric (BD biosciences, San Jose, CA, USA). Analyses were performed by using Flow jo 10.8.1software (BD biosciences) and the percentage of CD69-positive cells in the CD3-positive cell population after 24 h, as well as the percentage of CD25-positive cells in the CD3-positive cell population after 48 h were calculated.

### In vivo anti-tumor efficacy of 53L10 compared to 53 P and atezolizumab

The in vivo anti-tumor efficacy of the novel tribodies was evaluated in an A-549 (human alveolar basal epithelial adenocarcinoma cell line) xenograft mouse model. Human PBMCs (purchased from Cellular Technology Limited, Cleaveland, OH, USA) were activated by Dynabeads Human T-activator CD3/CD28 for 4 days prior to transplantation (activated PBMCs). A-549 lung cancer cells were subcutaneously transplanted into immunodeficient NOD/SCID mice with the same number of activated human PBMCs (Day 0). No special randomization methods were used. From the day of transplantation, administration of vehicle (PBS), 53 P (20 μg/mouse), or 53L10 (20 μg/mouse) into the tail vein was performed every other day for a total of 6 times (Days 0, 2, 4, 6, 8, and 10) in four groups of eight mice for each dose. Administration of atezolizumab (200 μg/mouse) into the tail vein was performed 3 times in total on Day 0, Day 4, and Day 8, in parallel to the combination of atezolizumab plus 53 P. Tumor volume was measured 3 days after transplantation and thereafter twice a week until 42 days after transplantation.

### Statistics methods

All the assays were conducted in three independent experiments carried out in triplicates. The data were shown as mean ± SD. The variation within each group of data was estimated to be lower than 10%. Analyses of lymphocytes were performed by using samples of hPBMCs obtained using at least three different donors. Statistical analyses for in vitro assays were assessed using Student’s *t* test (two variables). *p* ≤ 0.001***; *p* < 0.01**; and *p* < 0.05*, were considered statistically significant. Statistical analysis of tumor size differences on the final in vivo study day was evaluated by Tukey’s multiple comparison test using the statistical program MEPHAS (http://www.gen-info.osaka-u.ac.jp/MEPHAS/tukey-e.html) as a significance test: *P* < 0.05*.

## Supplementary information

Revised Supplementary Figures and legends

## Data Availability

All data generated or analyzed during this study are included in this published article [and its supplementary information files].
