# Supplementary material for: Tri-specific tribodies targeting 5T4, CD3, and immune checkpoint drive stronger functional T-cell responses than combinations of antibody therapeutics
Source: Cell Death Discov. 2025 Feb 10;11:58. doi: 10.1038/s41420-025-02329-8 (PMC11811032; doi:10.1038/s41420-025-02329-8)
Supplement: Supplementary file 1 — Revised Supplementary Figures and legends [file 41420_2025_2329_MOESM1_ESM.docx]

**TITLE:** [**Molecular insight in advantages of anti-tumor tri-specific tribodies with respect to combinations of clinically validated immunomodulatory mAbs**](https://pubmed.ncbi.nlm.nih.gov/17531523/) **with conventional T-cell engagers**

**AUTHORS**: Margherita Passariello^1,2^, Lorenzo Manna^1,2^, Rosa Rapuano Lembo^2,3^, Asami Yoshioka^4^, Toshikazu Inoue^4^, [Kentaro Kajiwara](mailto:kkajiwara@chiome.co.jp)^4^, Shu‑ichi Hashimoto^4^, Koji Nakamura^4^ and Claudia De Lorenzo^1,2^.

**AFFILIATIONS**:

**^1^** Department of Molecular Medicine and Medical Biotechnologies, University of Naples “Federico II”, 80131 Naples, Italy.

**^2^** Ceinge - Biotecnologie Avanzate S.C. a.R.L, via Gaetano Salvatore 486, 80145 Naples, Italy.

**^3^** European School of Molecular Medicine, University of Milan, 20122 Milan, Italy

**^4^** Chiome Bioscience Inc, 3‑12‑1 Honmachi Shibuya‑Ku, Tokyo 151‑0071, Japan. European School of Molecular Medicine, University of Milan, 20122 Milan, Italy

**CORRESPONDING AUTHOR**: Claudia De Lorenzo, email: [cladelor@unina.it](mailto:cladelor@unina.it), tel.: +39-081-3737868.

**Supplementary Figures and Legends**


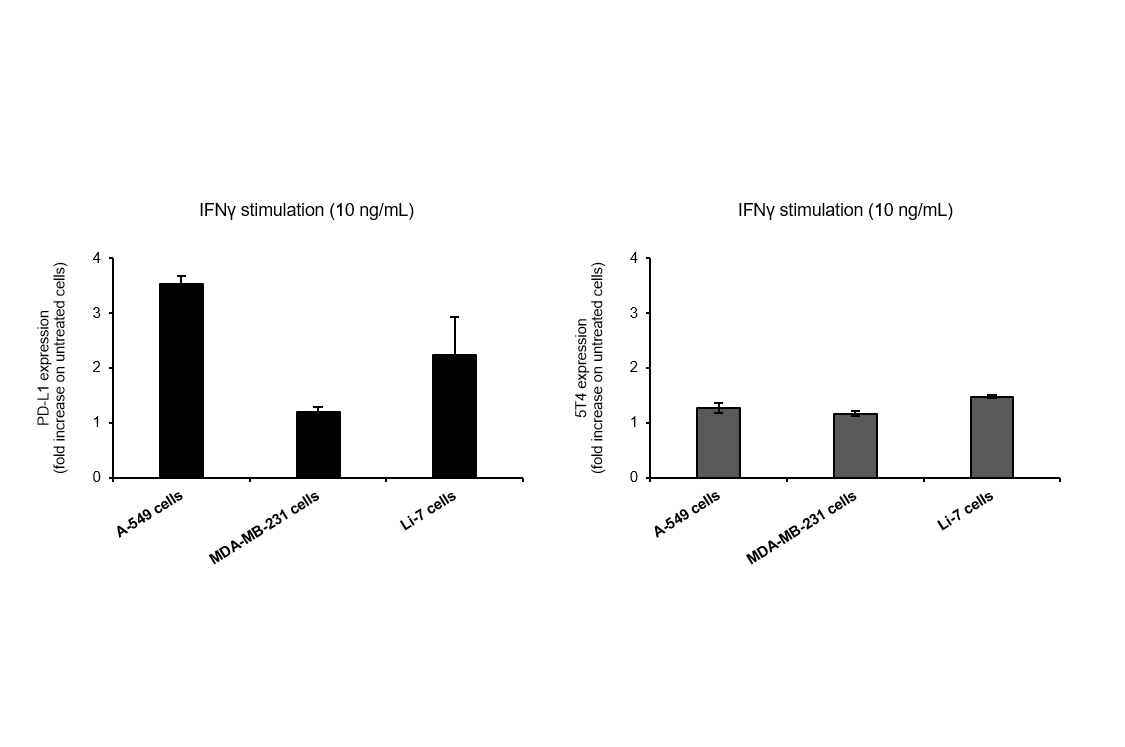


**Supplementary Fig. 1 Fold increase of PD-L1 and 5T4 expression after IFNγ stimulation on tumor cells**. Lung A-549, breast MDA-MB-231 and liver Li-7 cancer cells were stimulated with IFNγ used at the concentration of 10 ng/mL for 48 h and incubated at 37°C. Cell ELISA assays were performed to check the expression level of PD-L1 and 5T4 by using a commercial anti-PD-L1 or anti-5T4 antibody. Error bars depicted means ± SD.


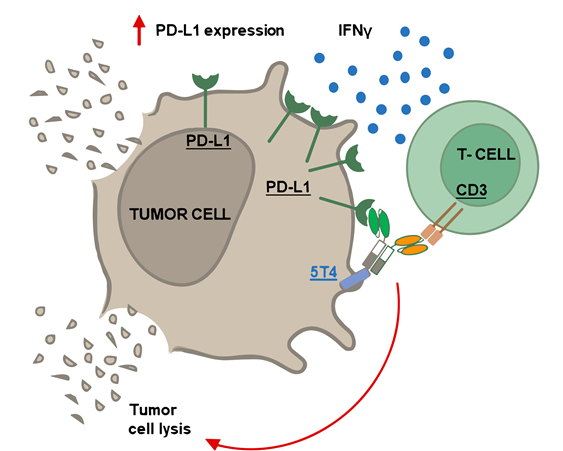


**Supplementary Fig. 2** **Model for the mechanism of action of anti-PD-L1 53L10 tri-specific tribody in tumor microenvironment.** Release of IFNγ induced by T cell activation promotes an increase of PD-L1 expression which leads to stronger binding and inhibition of PD-L1/PD-1 interaction by 53L10.


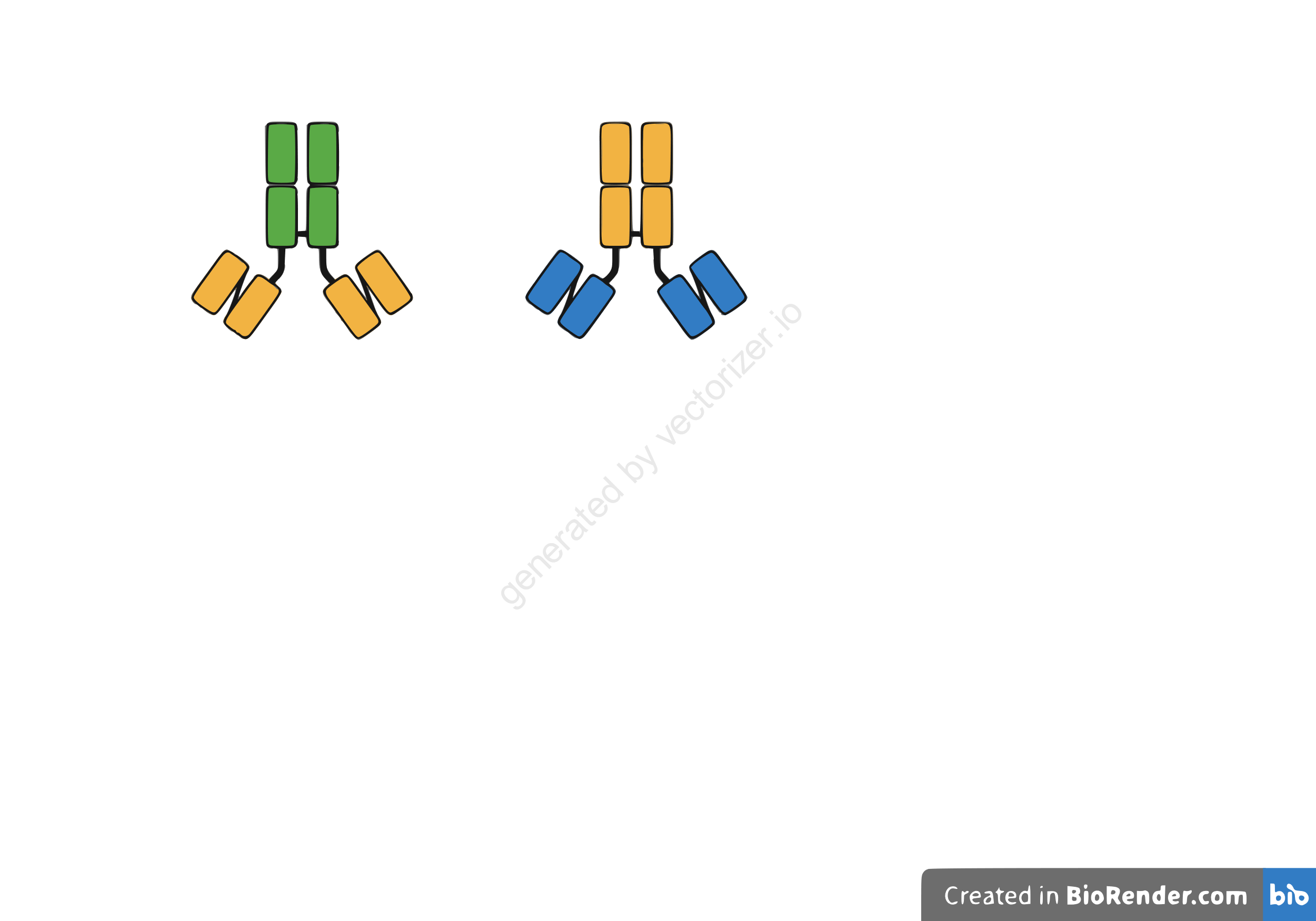


**α-PD-L1 Fab**

**α-LAG-3 scFv**

**α-LAG-3 scFv**

TR0304


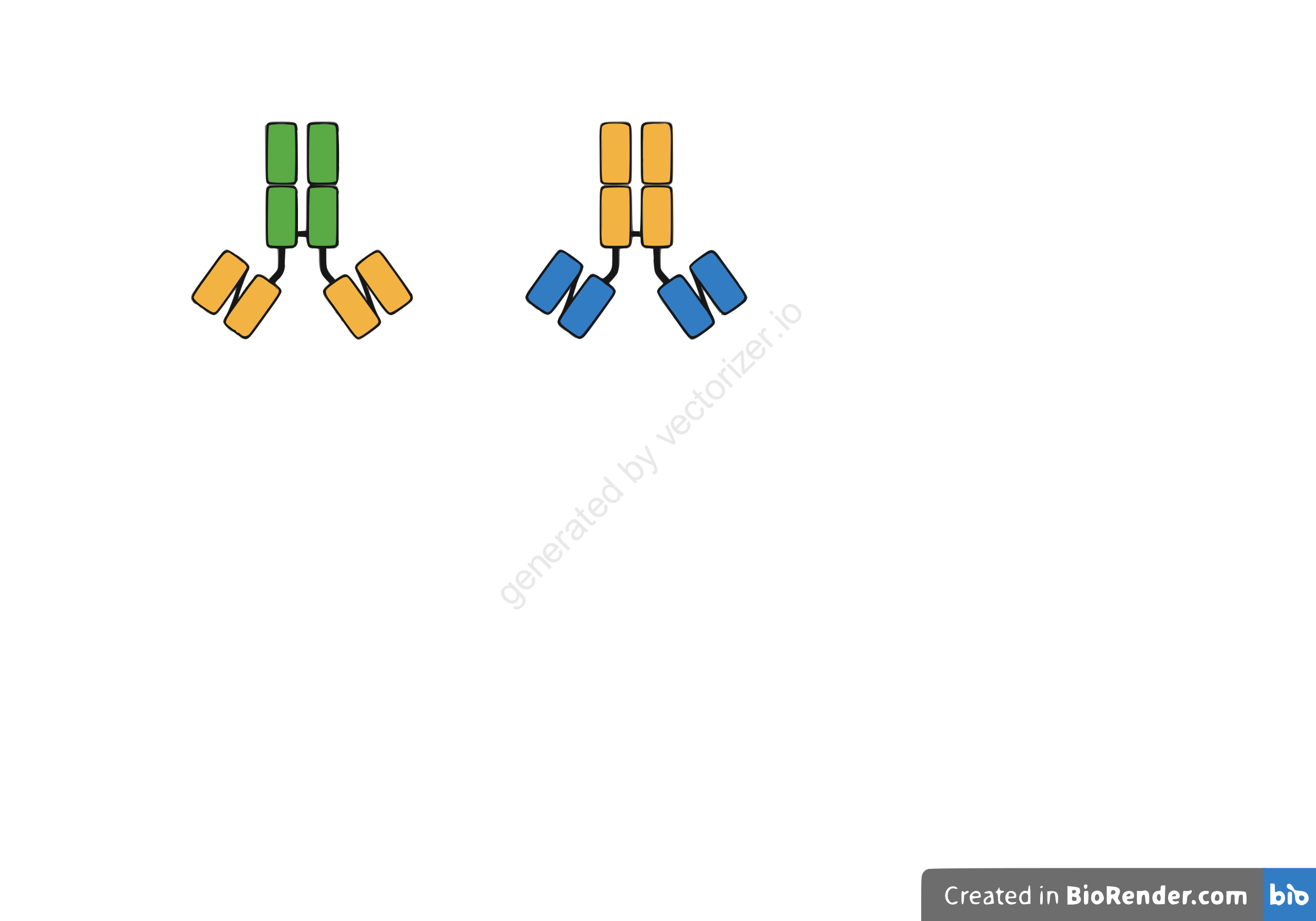


**α-PD-1 scFv**

**α-PD-1 scFv**

**α-LAG-3 Fab**

TR0506

**Supplementary Fig. 3 Graphical representation of the bi-specific tribodies 0304 and 0506.**The bi-specific TR0304 (left) and TR0506 (right) contain two scFvs, targeting LAG-3 or PD-1 respectively, genetically fused with a Fab specific for PD-L1 or LAG-3.
